# Plasmid Genomes Reveal the Distribution, Abundance, and Organization of Mercury-Related Genes and Their Co-Distribution with Antibiotic Resistant Genes in *Gammaproteobacteria*

**DOI:** 10.3390/genes13112149

**Published:** 2022-11-18

**Authors:** Xiangyang Li, Zilin Yang, Guohui Zhang, Shengli Si, Xianzhi Wu, Lin Cai

**Affiliations:** 1 School of Life and Health Science, Kaili University, Kaili 556011, China; 2 Bacterial Genome Data Mining & Bioinformatic Analysis Center, Kaili University, Kaili 556011, China; 3 School of Sciences, Kaili University, Kaili 556018, China; 4 Shenzhen Institute of Guangdong Ocean University, Shenzhen 518120, China

**Keywords:** plasmid, Mercury (Hg)-related genes (HRGs), antibiotic resistant genes (ARGs), co-distribution, *Gammaproteobacteria*

## Abstract

Mercury (Hg) pollution poses human health and environmental risks worldwide, as it can have toxic effects and causes selective pressure that facilitates the spread of antibiotic resistant genes (ARGs) among microbes. More and more studies have revealed that numerous Hg-related genes (HRGs) can help to resist and transform Hg. In the present study, we systematically analyzed the HRG distribution, abundance, organization, and their co-distribution with ARGs, using 18,731 publicly available plasmid genomes isolated from a *Gammaproteobacteria* host. Our results revealed that there were many Hg-resistant (*mer*) operon genes but they were not extensively distributed across plasmids, with only 9.20% of plasmids harboring HRGs. Additionally, no *hgcAB* genes (which methylate Hg to create methylmercury) were identified in any of the analyzed plasmids. The host source significantly influenced the number of HRGs harbored by plasmids; plasmids isolated from humans and animals harbored a significantly smaller number of HRGs than plasmids isolated from the wastewater and sludge. HRG clusters displayed an extremely high organizational diversity (88 HRG cluster types), though incidences of more than half of the HRG cluster types was <5. This indicates the frequent rearrangement among HRGs in plasmids. The 1368 plasmids harboring both HRGs and ARGs, were dominated by *Klebsiella*, followed by *Escherichia*, *Salmonella,* and *Enterobacter*. The tightness of the HRG and ARG co-distribution in plasmids was affected by the host sources but not by pathogenicity. HRGs were more likely to co-occur with specific ARG classes (sulfonamide, macrolide-lincosamide-streptogramin, and aminoglycoside resistance genes). Collectively, our results reveal the distribution characteristics of HRGs in plasmids, and they have important implications for further understanding the environmental risks caused by the spread of ARGs through the plasmid-mediated co-transfer of ARGs and HRGs.

## 1. Introduction

Mercury (Hg) occurs naturally in the Earth’s biogeochemical system. However, centuries of human activities, such as coal burning, mining, and other industrial activities, have massively increased the amounts of Hg in the atmosphere, ocean, and terrestrial systems [1]. Hg is a global environmental concern as its most chemical forms are toxic to all living organisms, especially methylmercury, which is a potent neurotoxin that affects human and animal development and health [2].

Microbes play an important role in the global geochemical cycle of Hg [3,4,5,6]. They have evolved various mechanisms to resist and transform Hg and organomercury compounds. These mechanisms include the reduction of Hg(II) to gaseous Hg(0), the oxidization of Hg(0) to Hg(II), the demethylation of organomercury compounds, and the methylation of Hg(II) [4,6,7,8,9]. Many microbial genes, which are denoted as Hg-related genes (HRGs) in this study, are implicated in these mechanisms. There is a growing consensus that there are more HRGs in microbial genomes than other metal-related genes.

The microbial detoxification of Hg mainly relies on the Hg-resistant (*mer*) operon, which has been reported to have originated in thermophiles [10,11]. Among the microbial metal resistant systems, only the *mer* operon detoxification mechanism leads to a large-scale transformation of its toxic target [4]. The *mer* operon can be located on transposons, plasmids, and chromosomes, and its components vary among the microbial genomes. The microbial *mer* genes include *merA* (encoding Hg(II) reductase), *merB* (encoding organomercury lyase), *merP* (encoding periplasmic Hg(II) scavenging protein), *merT, merC, merE, merF*, and *merG* (all encoding inner membrane-spanning proteins), and *merR* and merD (regulatory genes) [10]. Additionally, a two-gene cluster, *hgcA* and *hgcB*, is required for the Hg methylation in anaerobic microorganisms, typically in sulfur-reducing bacteria [9]. *hgcA* encodes a putative corrinoid protein with a strictly conserved cysteine that is proposed to be the ligand for the cobalt in the corrinoid cofactor, whereas *hgcB* encodes a ferredoxin-like protein, thought to be an electron donor to HgcA [9]. The *hgcAB* gene set has been found in nearly all anaerobic (but not aerobic) environments, including the oxygenated layers of the open ocean [12,13,14,15]. However, it has been reported to be effectively absent in mammalian microbiomes [12]. Furthermore, regarding the oxidization of Hg(0) to Hg(II) and the *mer*-independent oxidative demethylation, the biochemical pathways and the corresponding genes have not been identified [16].

Environmental Hg, which is directly toxic to living organisms, plays important roles in the spread of drug resistance among bacteria. Exposure to Hg and antibiotics has led to intersecting evolutionary pressures on bacteria, leading to cross-resistant bacterial phenotypes [17,18]. More and more studies have reported that bacteria from both clinical and natural environments can harbor a wealth of antibiotic resistant genes (ARGs) and HRGs, and these two types of genes are closely linked [19,20,21]. This may be explained by an adaptive evolution for bacterial genomes against Hg toxicity, and ancient antimicrobial agents before antibiotics were discovered for large production and use.

PCR- and metagenome- based methods have been extensively used to investigate the HRG distribution and the association of HRGs with AGRs [20]. However, the PCR-based approach can underestimate the abundance of HRGs when multiple copies of target genes are present in the microbial genomes. The metagenome- based approach can cause false positives due to the limitation regarding the short-read length. In most cases, neither PCR- nor metagenome-based methods can link specific genes to their respective hosts [22]. Therefore, the two approaches provide insufficient information to determine the distribution of HRGs and ARGs in individual genomes. 

Plasmids are highly transmissible genetic elements that play an important role in the spread of resistance genes [23,24]. However, our knowledge of the HRG distribution, abundance, organization, and co-distribution with ARGs in plasmids remains limited. So far, tens of thousands of plasmid genomes from the National Center for Biotechnology Information (NCBI) genome database were available, and more than half of the total available sequenced plasmids derived from a *Gammaproteobacteria* host. In this study, bioinformatics analyses were performed to annotate the HRGs and ARGs in plasmids isolated from a *Gammaproteobacteria* host. We then revealed the organizational diversity of HRGs, and the distribution of HRGs in plasmids isolated from various host sources. Additionally, we conducted an analysis of the physical genetic distances between ARGs and HRGs.

## 2. Materials and Methods

### 2.1. Retrieval of the Plasmid Genomic Sequences

All available plasmid genomes in *Gammaproteobacteria* (as of 6 December 2021) were retrieved from the NCBI genome database. Plasmids from the same strain that were sequenced more than once were randomly dereplicated. As a result, 18,731 plasmids were retained and annotated using a rapid prokaryotic sequence annotation algorithm implemented in Prokka v1.12 [25] to avoid any gene calling deviation. The assembly number and related information for all plasmid genomes are listed in Table S1.

The plasmid genetic location information, prokaryotic host, the taxonomic classification of the host, isolation source, and other information were retrieved from the original GenBank file in batches using an in-house Perl program. The protein-coding gene sequences of each plasmid were extracted from the GenBank files produced by Prokka and used for the HRG and ARG annotation. According to the “isolation source” and the “host information”, the host source of plasmids harboring HRGs were divided into the following categories: “human”, “animal”, “wastewater and sludge”, “miscellaneous sources”. The potential pathogenicity of each host species was obtained from the species list from the NCBI Pathogen Detection [26] and the published database covering the recognized species of human bacterial pathogens [27].

### 2.2. Annotation of HRGs

We constructed a HRG database by (1) extracting HRGs from BacMet version 2.0 [28] and (2) obtaining *hgcAB* gene sequences used by microorganisms to methylate Hg from previous studies [9,29,30,31]. First, utilizing an in-house Perl script, we used the keyword “Mercury” to search the “Compound” column of the BacMet2 gene annotation mapping file, to obtain the HRG GenBank IDs, which we used to extract the HRG sequences of the predicted HRGs in BacMet2. Second, the accession numbers or locus tags of the *hgcAB* genes from previous studies [9,29,30,31] were used to extract the *hgcAB* sequences from the NCBI protein database.

Our HRG database was then used to identify HRGs in each plasmid genome. We used the predicted proteomes as queries with the DIAMOND BLASTP [32]. Stringent criteria (e-value ≤ 1e-5; identity ≥ 80%; coverage ≥ 90%) [33,34] were used in each case, except for *hgcAB*, for which a lenient criterion (e-value ≤ 1e-5) that was used due to few similarities. Homologous *hgcA* sequences without the conserved sequence motif [G(I/V)NVWC(A/S/G)(A/S/G)GK] and the homologous *hgcB* sequences that were not near hgcA in the genomes were discarded [30,35].

### 2.3. Annotation of the ARGs

SARG version 2.0 is a structured ARG database that assigns query sequences to ARG classes, based on a similarity search [36]. We created an ARG database by using SARG as the primary database and integrating other sources of ARGs, comprising the Comprehensive Antibiotic Resistance Database (CARD) [37], ARGminder [38] and AMRFinderplus [39].

Each of these three databases was transformed into a SARG-like database by creating the following files: (1) a sequence file (FASTA format) involving a sequence ID (the ARG’s unique ID and ARG annotation separated by a blank), followed by the sequence data and (2) a list file (tab-delimited format) with two columns containing the following: (i) the ARG class and ARG name, separated by two consecutive underscores and (ii) each ARG’s unique ID (separated by a comma if there were multiple ARGs).

Next, the ARGs in the three databases were matched to the ARG class names used in the SARG database as follows: (1) if the ARG name in the list files of the three databases was identical to that in the SARG list file, the ARG class name (denoted X) was replaced (if it was not already the same) with that used in SARG (denoted Y) and (2) for all other ARGs that had this ARG class name (X), but a different ARG name from that in the SARG database, the ARG class name was replaced with that used in the SARG database (Y). Only 428 ARGs could not be matched to an ARG class name used in the SARG database, using the above steps. The ARG class of each of these ARGs was determined by searching them against the SARG database using DIAMOND BLASTP with a strict cutoff (e-value ≤ 1 − e^5^; identity ≥ 80%; coverage ≥ 90%) [34,40].

Our ARG database was then used to identify ARGs in each plasmid genome. We used the predicted proteomes of the plasmid genomes as queries, using the DIAMOND BLASTP algorithm with stringent criteria (e-value ≤ 1e-5; identity ≥ 80%; coverage ≥ 90%) [34,40].

### 2.4. Co-Distribution Analysis of the ARGs and HRGs

Plasmids harboring both ARGs and HRGs were used for a co-distribution analysis. Based on the method used in a previous study [34], two parameters were used to accurately analyze the co-distribution of ARGs and HRGs in plasmids. The two parameters were The A-H-incidence_ave_ (the average number of ARGs that occur within a specified physical genetic distance of HRGs among all plasmids) and the A-H-distance_min_ (the minimum distance between ARGs and HRGs in each plasmid) [34].

The A-H-incidence_ave_ was calculated as follows: (1) the physical genetic distance between each ARG–HRG pair in each plasmid was calculated, (2) the cumulative number of ARGs within the specified distance (distance increased from 0 to 100 kb with 200 bp as the step width) was recorded for each plasmid, and (3) the A-H-incidence_ave_ was obtained by dividing the total number of ARGs within the specified physical genetic distance of HRGs by the total number of plasmids.

The A-H-distance_min_ was calculated as follows: (1) the physical genetic distance between each ARG–HRG pair in each plasmid was calculated, (2) the HRGs were subjected to de-redundancy, ensuring that each HRG class appeared only once, and (3) the A-H-distance_min_ was obtained by determining the minimum distance between an ARG and a HRG, for each ARG class in each plasmid.

### 2.5. Analysis of Preference of the HRGs for Specific ARG Classes

In step 3 of the calculation of the A-H-distance_min_, the minimum distance between any ARGs and HRGs in each plasmid was obtained.

### 2.6. Statistical Analysis

A Kruskal–Wallis test, a Wilcoxon rank-sum test, and plotting were performed in R software version 4.1.3 (R Core Team Vienna, Austria) with the “ggpubr” package. A result was considered significant if *p* < 0.05, highly significant if *p* < 0.01, and extremely significant if *p* < 0.001, according to the previous studies.

## 3. Results and Discussion

### 3.1. Overview of the Plasmid Host Taxonomy

According to the host taxonomic classification, all of the 18,731 plasmids affiliated to 135 genera, but 93.09% of the plasmid numbers were concentrated in 17 genera host (Table S2). The predominant genera were *Escherichia* and *Klebsiella*, and they occupied 54.57% of all plasmids belonged to these two genera. It is important to note that although the large number of plasmids used in this study led to a relatively comprehensive HRG profile, they do not necessarily represent the complete microbial diversity in *Gammaproteobacteria* [34].

### 3.2. Distribution of the HRGs in Plasmid Genomes

**Figure 1:**
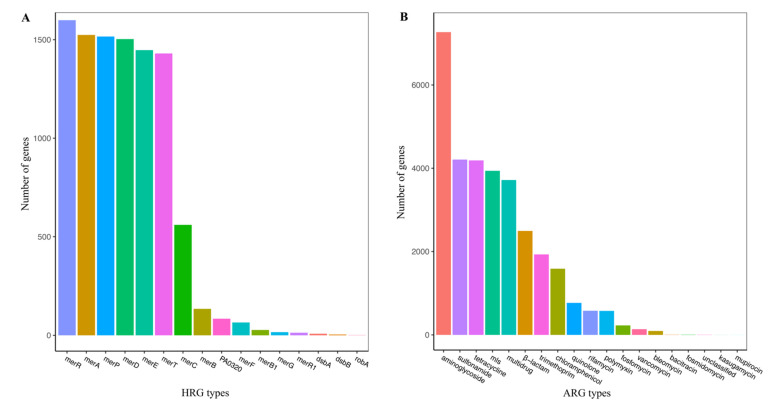
Distribution of the HRG (**A**) and ARG (**B**) classes in all analyzed plasmids. mls: macrolide−lincosamide−streptogramin.

To ascertain the distribution and organizational diversity of the HRGs, all plasmids were analyzed, based on a sequence similarity search analysis. The results indicated that 1723 plasmids harbored HRGs, accounting for only 9.20% of all plasmids. In these plasmids, 9931 HRGs, belonging to 16 HRG classes, were identified (Figure 1A). There were many HRGs but they were not extensively distributed across the plasmids.

The most common HRGs were *merR*, *merA*, *merP*, *merD*, *merE*, and *merT*. They were similar in number, reflecting the relatively high prevalence of HRG clusters, composed of these six HRGs in plasmids. The number of HRGs in each plasmid was highly variable. The maximum number of HRGs (24) was found in both plasmid pF10AN_1 (305.55 kp) from the *Klebsiella pneumoniae* strain F10(AN) and plasmid unnamed1 (150.42 kp) from the *Enterobacter cloacae* complex sp. strain AR_0072 (Table S3). The genome size of the 1723 plasmids that harbored HRGs varied markedly, from 5.37 to 1039.05 kb. Inevitably, larger plasmids had more genes, there was a certain degree of correlation between the plasmid genome size and the number of HRGs (Spearman’s ρ = 0.30, *p*-value < 2.2e^−16^). 

The hosts of the plasmids harboring HRGs belonged to 32 genera, dominated by *Klebsiella*, *Escherichia*, *Salmonella*, *Enterobacter,* and *Citrobacter*, all of which are potential pathogens (Table 1). These five genera accounted for more than 83% of the total number of plasmids carrying HRGs. Thus, HRGs were frequently associated with pathogenic strains. It might be a coincidence that the first reported bacterium that exhibited Hg resistance was a clinical isolate, which was also penicillin resistant [41]. However, it is likely that many more plasmids were isolated from pathogens than non-pathogens because there has been more medical microbiology research conducted by microbiologists. As for five genera (*Xanthomonas*, *Piscirickettsia*, *Vibrio*, *Erwinia,* and *Yersinia*), the number of plasmids from each of the genera was greater than 100, but there was none with or less than 1% of HRGs identified in the plasmids. There may be a distribution preference for HRGs related to plasmid hosts at the genus level. For instance, out of 5508 plasmids isolated from *Escherichia*, only 267 plasmids carried HRGs, while, 139 out of 655 plasmids from *Citrobacter* contained HRGs.

The microbial *hgcAB* genes used to methylate Hg have been found in highly diverse anaerobic settings, e.g., soils, sediments, rice paddies, and various extreme environments [9,12,35,42]. However, the *hgcAB* gene set is relatively rare, occurring in only ~1.4% of the sequenced microbial genomes [12]. In the present study, using a fairly lenient criterion (e-value ≤ 1−e^5^) plus a conserved *hgcA* sequence motif search, the *hgcAB* gene set was not identified in any plasmids. Thus, the *hgcAB* gene set appears to be absent from plasmids, and is more likely to be located in microbial chromosomes, suggesting a low risk of a *hgcAB* transfer.

**Figure 2:**
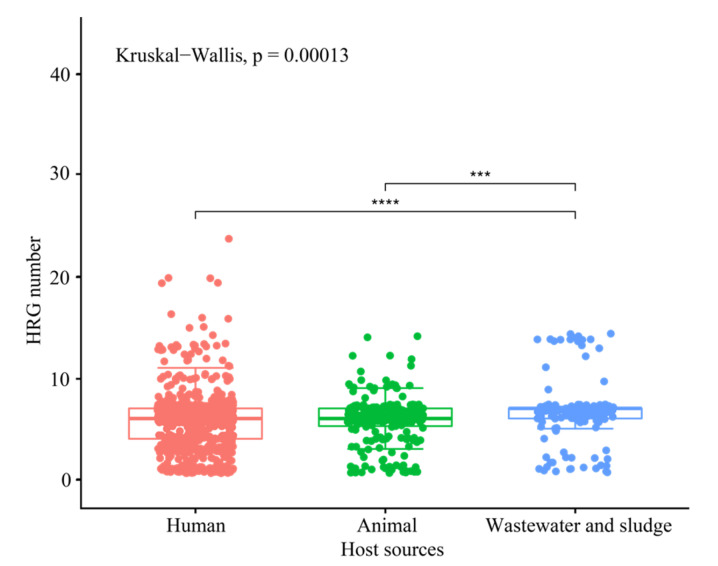
Number of HRGs per plasmid by the host source, showing that the host source impacts the distribution of HRGs in plasmids. *** *p* < 0.001 and **** *p* < 0.0001 based on Wilcoxon rank-sum test after a significant Kruskal–Wallis.

According to the plasmid host source, they were classified into several groups: human (901 plasmids), animal (242 plasmids), wastewater and sludge (144 plasmids), and miscellaneous sources (159 plasmids). The host source for 277 plasmids were unknown (denoted as NA in Table S3). Overall, there were significant differences in the number of HRGs in the plasmids isolated from these different sources (Figure 2). Specifically, the median numbers of HRGs in plasmids isolated from humans (six HRGs), animals (six HRGs), both of which are slightly smaller than that of the isolates from the wastewater and sludge (seven HRGs) (Figure 2). It accorded with a previous study, reported by us, that the number of arsenic-related genes per *Burkholderiales* isolate from humans and animals was less than that of the isolates from wastewater and sludge [22]. Plasmid from human and animal hosts have a median of HRGs up to six. However, we found no significant difference in the number of HRGs between the plasmids isolated from humans and animals. We speculate that our results may be related to the fact that before antibiotic use, Hg compounds were extensively used as disinfectants and antiseptics in hospitals and communities. Nevertheless, our results revealed that the host source plays a crucial role in influencing the distribution of HRGs in plasmids.

### 3.3. HRG Clusters Are Highly Abundant and Have an Extremely High Organizational Diversity

The bacterial *mer* operon plays a crucial role in the Hg biogeochemistry and bioremediation by converting both the reactive inorganic and organic forms of Hg to relatively inert, volatile, and monoatomic forms [43]. Consistent with previous studies [4,44], almost all of the identified HRGs (96.29%) were grouped into clusters, which indicates that HRGs tend to work together. To investigate the organizational diversity of the HRG clusters (referring to *mer* clusters), we defined a HRG cluster as at least two HRGs that occur together or are separated by no more than two other genes. We thereby identified 88 HRG cluster types, with 1680 incidences in plasmids (Table 2).

merR-merT-merP-merC/X-merA-merD-merE represent the two most common HRG cluster types, together accounting for 59.52% of all incidences of clusters (Table 2). Of the 88 HRG cluster types, 64 (72.73%) had an incidence of <5. Taken together, compared to the small number of HRG classes (16 HRG classes), the HRG cluster types are extremely abundant and diverse [4]. This phenomenon further highlights the frequent rearrangement that occurs among HRGs [4]. The extensive findings regarding HRGs in this study enrichened the gene sources for the exploitation of the mer-mediated functions for the Hg bioremediation [44]. Along with the fairly high diversity of HRG clusters, the transcription of HRGs can be activated in diverse ways, e.g., the merA transcription activation in Thermus thermophilus [45].

### 3.4. Co-Distribution of HRGs and ARGs in Plasmid Genomes

Of the 18,731 plasmids, 6182 harbored ARGs, accounting for 24.01%. These plasmids harbored 31,723 ARGs, which were related to a resistance against 19 broad classes of antibiotics (Figure 1B). The predominant ARG classes were related to a resistance against aminoglycosides (22.91%), sulfonamides (13.26%), tetracycline (13.20%), and macrolide-lincosamide-streptomycin (12.41%).

**Figure 3:**
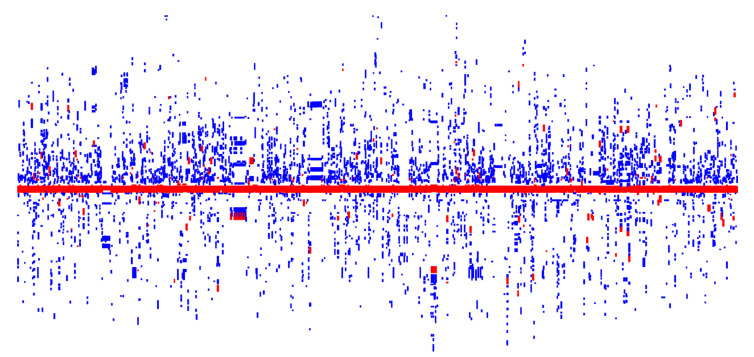
Visualization of the co-distribution of HRGs and ARGs in plasmids. Each plasmid is denoted by a vertical column, and the length of the HRGs (red), ARGs (blue), and all the other genes (white) represent the gene length. A maximum of 200 genes flanking the *merA* gene (in red) of each plasmid are visualized using Gcluster [47].

Many studies have reported finding the HRG and ARG co-distribution in various complex environments or in single microorganisms [20,21,24,45,46]. We found that there were 1368 plasmids harboring both HRGs and ARGs, representing approximately 7.30%, 79.40%, and 22.13% of all analyzed plasmids, plasmids with HRGs, and plasmids with ARGs, respectively (Table S4). The plasmid hosts belonged to 25 genera, but were largely concentrated in *Klebsiella*, *Escherichia*, *Salmonella,* and *Enterobacter* (Table 1). The number of HRGs and ARGs harbored by the 1368 plasmids were 8146 and 10,207, accounting for 82.03% and 32.18% of the HRGs (out of a total of 9931) and ARGs (out of a total of 31,723) of all analyzed plasmids, respectively. In some of the plasmids that harbored both HRGs and ARGs, HRGs were flanked by numerous ARGs (Figure 3). In contrast to the ratio of the co-distribution plasmid number in plasmids with HRGs (79.40%) and plasmids with ARGs (22.13%), the plasmids harboring both ARGs and HRGs had a high ratio of resistance genes, HRGs (82.03%) and ARGs (32.18%). Thus, ARGs and HRGs tend to be co-distributed. Furthermore, the incidences of ARGs and HRGs were slightly positively correlated (Spearman’s ρ = 0.23, *p*-value < 2.2e^−16^).

We speculate that the correlation between ARGs and HRGs might be attributable to a co-selection mechanism [48]. For instance, at a water treatment plant that disinfected surface water, the number of HRGs and ARGs related to multidrug, β-lactam, and aminoglycoside resistance increased [46]. Evolutionary events that lead to the emergence of new microbial antibiotic resistance factors are rare, while transmission events of widespread resistance genes are common [48]. As key drivers of horizontal gene transfer, plasmids harboring both HRGs and ARGs can be transferred at seemingly high rates via several mechanisms [49]. For example, Hg pollution in soil accelerates the transfer of ARGs among bacteria [50,51].

**Figure 4:**
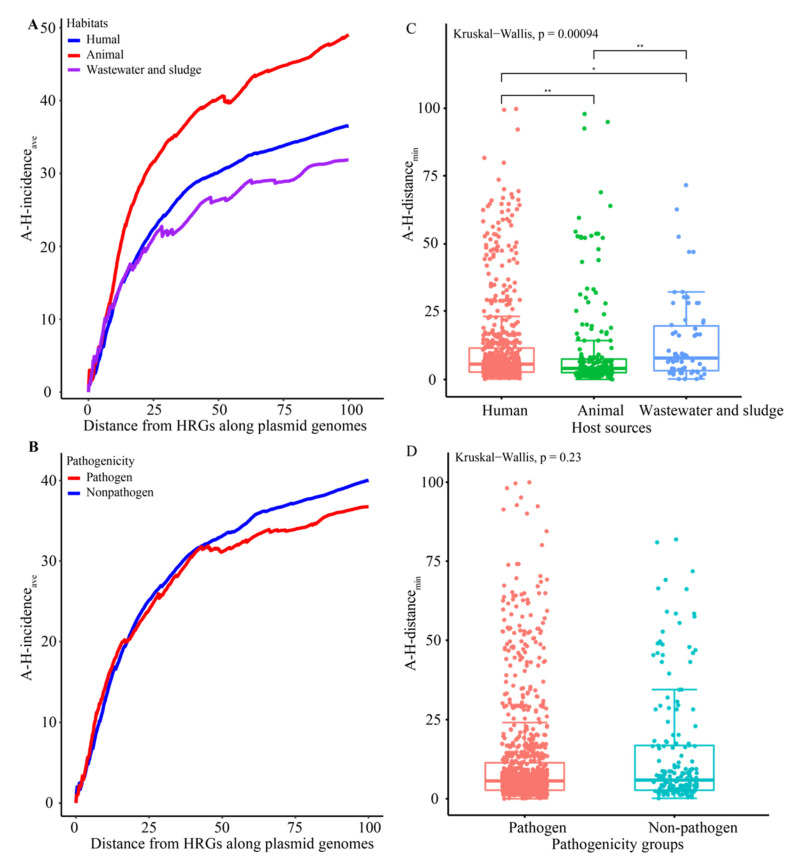
ARG and HRG co-distribution by the host source (**A**,**C**) and pathogenicity (**B**,**D**). (**A**,**B**) Change in the A-H-incidence_ave_ (the average number of ARGs within a specified distance of HRGs among all plasmids with an increased distance. (**C**,**D**) A-H-distance_min_ (average minimum distance between ARGs and HRGs among all plasmids. * *p* < 0.05 and ** *p* < 0.01 based on Wilcoxon rank-sum test after a significant Kruskal–Wallis.

We also analyzed the physical genetic distance between the co-distributed HRGs and ARGs using the A-H-incidence_ave_ and the A-H-distance_min_ (see “*Co-Distribution Analysis of the ARGs and HRGs*” section in Section 2.4). The results indicated that ARGs were located very close to HRGs, with a median A-H-distance_min_ of 5.67 kb. As the distance from the HRGs increased, the A-H-incidence_aves_ tended to be greater in plasmids from humans and animals than plasmids from wastewater and sludge (Figure 4A). Furthermore, based on the A-H-distance_min_, ARGs and HRGs were much closer in plasmids from humans (a median A-H-distance_min_ of 5.67 kb) and animals (a median A-H-distance_min_ of 4.10 kb) than in plasmids from wastewater and sludge (a median A-H-distance_min_ of 8.46 kb) (Figure 4C). This is consistent with a previous report showing that bacteria from humans and animals had the highest co-selection potential [52]. This may be due to humans and animals being the main bacterial host sources that involve a regular and intentional high exposure to antibiotics. Thus, the high concentrations of antibiotics drove the increased ARG and HRG co-distribution [52].

As the distance from HRGs increased, a rising trend of the A-H-incidence_aves_ increased more in the plasmids from pathogens, compared to the plasmids from non-pathogens (Figure 4B). While, there was no significant difference on the physical genetic distance between ARGs and HRGs for the plasmids from pathogens (a median A-H-distance_min_ of 5.67 kb) and non-pathogens (a median A-H-distance_min_ of 5.92 kb) (Figure 4D). These results hint that the occurrences of HRGs and ARGs are closely related in pathogens, as is the case in a previous study of complete bacterial genomes [34]. The degree of difference is more obvious in that study [34]. We speculate that the shuttling of plasmids harboring both ARGs and HRGs between clinical and non-clinical hosts weakened the difference between the pathogens and non-pathogens.

### 3.5. Preference of HRGs for Specific ARG Classes

In microbial genomes, functionally related genes (e.g., functional genes conferring resistance to specific antibiotics and metals) tend to cluster together, becoming adjacent to each other due to selective pressure. Based on the physical genetic distance calculations, the nearest ARG classes to the HRGs were analyzed to reveal the functional association between the ARG classes and HRGs. HRGs had a clear preference for several ARG classes (related to a resistance against sulfonamide, macrolide-lincosamide-streptogramin, aminoglycoside, tetracycline, and β-lactam), as they were close in terms of the physical genetic position (Table 3). Conclusively, the empirical observation of the genetic co-distribution (Figure 3) indicates that Hg exposure can promote resistance against a wide range of antibiotics, and the selective preference for particular classes of antibiotics is obvious [52]. As the formation and spread of drug resistance (especially in bacterial pathogens) is a severe health issue, which leads to millions of human deaths each year, more attention should be paid to the co-transmission of ARGs with HRGs caused by the Hg selective pressure.

## 4. Conclusions

In this study, a bioinformatics analysis of a large number of plasmid genomes confirmed that there were many HRGs but they were not extensively distributed across the plasmids, with only 9.20% of plasmids harboring HRGs. We found that the HRG clusters had a high organizational diversity, with 88 HRG cluster types being found in plasmids, despite the relatively small number of HRG types. Moreover, the incidences of more than half of the HRG cluster types were low. These results suggest that HRG clusters in plasmids were subjected to the frequent gene rearrangement, in response to the extensive historical use of Hg. Narrow-spectrum Hg-resistance operons were the predominant HRG cluster types in plasmids, mainly involving *merR*-*merT*-*merP*-*merC/X*-*merA*-*merD*-*merE*. Fortunately, the *hgcAB* gene set (which confers the ability to methylate Hg(II)) was not present in the plasmids, which lowers the risk of a methylmercury release into the environment. A substantial proportion of plasmids harboring HRGs also harbored ARGs (79.40%), and plasmids harboring both ARGs and HRGs had higher ratios of HRGs and ARGs than those of all analyzed plasmids. Furthermore, there was a weak but significant correlation between HRGs and ARGs. Thus, we speculate that HRGs and ARGs are prone to co-distribution in plasmids. In addition, the HRG and ARG distribution in plasmids was related to the host source. Plasmids from humans and animals, compared to wastewater and sludge, had a greater increase in the incidence of ARGs within a specified physical genetic distance of HRGs as the specified distance increased, and a smaller average minimum distance between ARGs and HRGs, among all plasmids harboring both ARGs and HRGs. These findings suggest that the plasmid hosts exposed to antibiotics or Hg, might acquire HRGs and ARGs during the long-term adaptive evolution under selective pressure. In summary, this study highlighted the incidence of ARGs (which pose large health risks) caused by the environmental Hg contamination, which has resulted in the frequent plasmid-mediated transfer of ARGs and HRGs. It should be noted that the findings should not be extended too far into the environment, since the skew in the data has its roots in our interest in medically relevant pathogens.

## Figures and Tables

**Table 1:** Taxonomic distribution of HRGs and ARGs in plasmid hosts.

Genus *	All Plasmids		Plasmids Harboring HRGs		Plasmids Harboring Both HRGs and ARGs	
	Plasmid Number	Percent (%)	Plasmid Number	Percent (%)	Plasmid Number	Percent (%)
*Escherichia*	5508	29.41	267	15.50	258	18.86
*Klebsiella*	4714	25.17	614	35.64	542	39.62
*Acinetobacter*	1438	7.68	58	3.37	19	1.39
*Salmonella*	1343	7.17	249	14.45	178	13.01
*Enterobacter*	1031	5.50	175	10.16	150	10.96
*Citrobacter*	655	3.50	139	8.07	75	5.48
*Xanthomonas*	398	2.12	1	0.06	1	0.07
*Pseudomonas*	385	2.06	75	4.35	38	2.78
*Shigella*	288	1.54	4	0.23	3	0.22
*Vibrio*	258	1.38	1	0.06	1	0.07
*Aeromonas*	225	1.20	29	1.68	25	1.83
*Pantoea*	197	1.05	4	0.23	4	0.29
*Yersinia*	187	1.00	1	0.06	1	0.07
*Serratia*	149	0.80	17	0.99	12	0.88
*Raoultella*	113	0.60	16	0.93	11	0.80
*Proteus*	81	0.43	10	0.58	10	0.73
*Providencia*	77	0.41	6	0.35	6	0.44
Unclassed genera	58	0.31	7	0.41	6	0.44
*Moraxella*	54	0.29	4	0.23		
*Cronobacter*	52	0.28	2	0.12	2	0.15
*Shewanella*	47	0.25	8	0.46	6	0.44
*Edwardsiella*	38	0.20	1	0.06	1	0.07
*Leclercia*	37	0.20	12	0.70	12	0.88
*Pseudoalteromonas*	25	0.13	2	0.12		
*Alteromonas*	19	0.10	3	0.17		
*Halomonas*	14	0.07	4	0.23		
*Marinobacter*	11	0.06	2	0.12		
*Stenotrophomonas*	10	0.05	4	0.23	2	0.15
*Kluyvera*	7	0.04	2	0.12	1	0.07
*Phytobacter*	5	0.03	3	0.17	3	0.22
*Mixta*	3	0.02	1	0.06	1	0.07
*Marinomonas*	1	0.01	1	0.06		
*Salinimonas*	1	0.01	1	0.06		

* Genus was not shown if the plasmid for this host had no HRG.

**Table 2:** Diversity of the HRG cluster types in plasmid genomes.

HRG Cluster Type	Incidence *	Percent of Incidences (%)
*merR-merT-merP-X-merA-merD-merE*	543	32.32
*merR-merT-merP-merC-merA-merD-merE*	457	27.20
*merA-merD-merE*	96	5.71
*merR-merT-merP-merA-merB-merD-merE*	77	4.58
*merR-merT-merP-X-merA*	76	4.52
*merR-merT-merP-merF-merA-merD-merE*	62	3.69
*merD-merE*	48	2.86
*merR-merT-merP-merA-merD-merE*	39	2.32
*merR-merT-merP*	31	1.85
*merR-X-merB-merD-merE*	27	1.61
*merR-merT-merP-X-merA-merD*	16	0.95
*merR-X-X-merC-merA-merD*	16	0.95
*merR-merT-merP-merC-merA-merD*	14	0.83
*merR-merT*	11	0.65
*merR-merT-merP-merC-merA*	10	0.60
*merR-merT-merP-merA-merG-merB1*	9	0.54
*merR-merT-merP-merA-merB-X-X-merE*	8	0.48
*merT-merP-X-merA-merD-merE*	7	0.42
*merP-X-merA*	6	0.36
*merR-X-X-merC-X-X-merD*	6	0.36
*merT-merP-merC-merA-merD-merE*	5	0.30
*merP-X-merA-merD-merE*	5	0.30
*merB-merD-merE*	5	0.30
*merR-X-merC-merA*	5	0.30

* HRG cluster types with an incidence <5 are not shown.

**Table 3:** Preferences of HRGs for various ARG classes (excluding multidrug resistant genes) in plasmid genomes.

ARG Class Nearest to HRGs	Plasmid Number	Percentage (%)
sulfonamide	351	26.77
macrolide-lincosamide-streptogramin	332	25.32
aminoglycoside	195	14.87
tetracycline	154	11.75
β-lactam	126	9.61
chloramphenicol	53	4.04
trimethoprim	33	2.52
quinolone	26	1.98
polymyxin	20	1.53
vancomycin	11	0.84
fosfomycin	6	0.46
rifamycin	3	0.23
kasugamycin	1	0.08

## Data Availability

All plasmid genomes in this study are openly available on the NCBI. Database, and the accession numbers for all plasmid genomes are provided in Table S1.

## Supplementary Materials

The following supporting information can be downloaded at: https://www.mdpi.com/article/10.3390/genes13112149/s1, Table S1: Plasmid genomes used in this study, Table S2: Taxonomic distribution of the plasmid hosts, Table S3: The hosts, pathogenicity, and number of HRGs for 2006 plasmids, and Table S4: Co-distribution of HRGs and ARGs in each Plasmid.

## References

[1] Selin N.E. (2009). Global Biogeochemical Cycling of Mercury: A Review. Annu. Rev. Environ. Resour..

[2] Mergler D., Anderson H.A., Chan L.H., Mahaffey K.R., Murray M., Sakamoto M., Stern A.H., Panel on Health R., Toxicological Effects of M. (2007). Methylmercury exposure and health effects in humans: A worldwide concern. Ambio.

[3] Lu X., Liu Y., Johs A., Zhao L., Wang T., Yang Z., Lin H., Elias D.A., Pierce E.M., Liang L. (2016). Anaerobic Mercury Methylation and Demethylation by Geobacter bemidjiensis Bem. Environ. Sci. Technol..

[4] Barkay T., Miller S.M., Summers A.O. (2003). Bacterial mercury resistance from atoms to ecosystems. FEMS Microbiol. Rev..

[5] Petrus A.K., Rutner C., Liu S., Wang Y., Wiatrowski H.A. (2015). Mercury Reduction and Methyl Mercury Degradation by the Soil Bacterium Xanthobacter autotrophicus Py2. Appl. Environ. Microbiol..

[6] Colombo M.J., Ha J., Reinfelder J.R., Barkay T., Yee N. (2014). Oxidation of Hg(0) to Hg(II) by diverse anaerobic bacteria. Chem. Geol..

[7] Natasha, Shahid M., Khalid S., Bibi I., Bundschuh J., Khan Niazi N., Dumat C. (2020). A critical review of mercury speciation, bioavailability, toxicity and detoxification in soil-plant environment: Ecotoxicology and health risk assessment. Sci. Total Environ..

[8] Oremland R.S., Culbertson C.W., Winfrey M.R. (1991). Methylmercury decomposition in sediments and bacterial cultures: Involvement of methanogens and sulfate reducers in oxidative demethylation. Appl. Environ. Microbiol..

[9] Parks J.M., Johs A., Podar M., Bridou R., Hurt R.A., Smith S.D., Tomanicek S.J., Qian Y., Brown S.D., Brandt C.C. (2013). The genetic basis for bacterial mercury methylation. Science.

[10] Boyd E.S., Barkay T. (2012). The mercury resistance operon: From an origin in a geothermal environment to an efficient detoxification machine. Front. Microbiol..

[11] Barkay T., Kritee K., Boyd E., Geesey G. (2010). A thermophilic bacterial origin and subsequent constraints by redox, light and salinity on the evolution of the microbial mercuric reductase. Environ. Microbiol..

[12] Podar M., Gilmour C.C., Brandt C.C., Soren A., Brown S.D., Crable B.R., Palumbo A.V., Somenahally A.C., Elias D.A. (2015). Global prevalence and distribution of genes and microorganisms involved in mercury methylation. Sci. Adv..

[13] Gionfriddo C.M., Stott M.B., Power J.F., Ogorek J.M., Krabbenhoft D.P., Wick R., Holt K., Chen L.X., Thomas B.C., Banfield J.F. (2020). Genome-Resolved Metagenomics and Detailed Geochemical Speciation Analyses Yield New Insights into Microbial Mercury Cycling in Geothermal Springs. Appl. Environ. Microbiol..

[14] Jones D.S., Johnson N.W., Mitchell C.P.J., Walker G.M., Bailey J.V., Pastor J., Swain E.B. (2020). Diverse Communities of hgcAB(+) Microorganisms Methylate Mercury in Freshwater Sediments Subjected to Experimental Sulfate Loading. Environ. Sci. Technol..

[15] Peterson B.D., McDaniel E.A., Schmidt A.G., Lepak R.F., Janssen S.E., Tran P.Q., Marick R.A., Ogorek J.M., DeWild J.F., Krabbenhoft D.P. (2020). Mercury Methylation Genes Identified across Diverse Anaerobic Microbial Guilds in a Eutrophic Sulfate-Enriched Lake. Environ. Sci. Technol..

[16] Barkay T., Gu B. (2021). Demethylation─The Other Side of the Mercury Methylation Coin: A Critical Review. ACS Environ. Au.

[17] Imran M., Das K.R., Naik M.M. (2019). Co-selection of multi-antibiotic resistance in bacterial pathogens in metal and microplastic contaminated environments: An emerging health threat. Chemosphere.

[18] Lloyd N.A., Janssen S.E., Reinfelder J.R., Barkay T. (2016). Co-selection of Mercury and Multiple Antibiotic Resistances in Bacteria Exposed to Mercury in the Fundulus heteroclitus Gut Microbiome. Curr. Microbiol..

[19] Skurnik D., Ruimy R., Ready D., Ruppe E., Bernede-Bauduin C., Djossou F., Guillemot D., Pier G.B., Andremont A. (2010). Is exposure to mercury a driving force for the carriage of antibiotic resistance genes?. J. Med. Microbiol..

[20] Yuan L., Li Z.H., Zhang M.Q., Shao W., Fan Y.Y., Sheng G.P. (2019). Mercury/silver resistance genes and their association with antibiotic resistance genes and microbial community in a municipal wastewater treatment plant. Sci. Total Environ..

[21] Gaeta N.C., de Carvalho D.U., Fontana H., Sano E., Moura Q., Fuga B., Munoz P.M., Gregory L., Lincopan N. (2022). Genomic features of a multidrug-resistant and mercury-tolerant environmental Escherichia coli recovered after a mining dam disaster in South America. Sci. Total Environ..

[22] Li X., Zhang L., Wang G. (2014). Genomic evidence reveals the extreme diversity and wide distribution of the arsenic-related genes in Burkholderiales. PLoS ONE.

[23] Kothari A., Soneja D., Tang A., Carlson H.K., Deutschbauer A.M., Mukhopadhyay A. (2019). Native Plasmid-Encoded Mercury Resistance Genes Are Functional and Demonstrate Natural Transformation in Environmental Bacterial Isolates. mSystems.

[24] Perez-Palacios P., Delgado-Valverde M., Gual-de-Torrella A., Oteo-Iglesias J., Pascual A., Fernandez-Cuenca F. (2021). Co-transfer of plasmid-encoded bla carbapenemases genes and mercury resistance operon in high-risk clones of Klebsiella pneumoniae. Appl. Microbiol. Biotechnol..

[25] Seemann T. (2014). Prokka: Rapid prokaryotic genome annotation. Bioinformatics.

[26] Organism Groups. https://www.ncbi.nlm.nih.gov/pathogens/organisms/.

[27] Woolhouse M.E., Gowtage-Sequeria S. (2005). Host range and emerging and reemerging pathogens. Emerg. Infect. Dis..

[28] Pal C., Bengtsson-Palme J., Rensing C., Kristiansson E., Larsson D.G. (2014). BacMet: Antibacterial biocide and metal resistance genes database. Nucleic Acids Res..

[29] Liu Y.R., Johs A., Bi L., Lu X., Hu H.W., Sun D., He J.Z., Gu B. (2018). Unraveling Microbial Communities Associated with Methylmercury Production in Paddy Soils. Environ. Sci. Technol..

[30] Zhang L., Philben M., Tas N., Johs A., Yang Z., Wullschleger S.D., Graham D.E., Pierce E.M., Gu B. (2022). Unravelling biogeochemical drivers of methylmercury production in an Arctic fen soil and a bog soil. Environ. Pollut..

[31] Liu J., Li Y., Duan D., Peng G., Li P., Lei P., Zhong H., Tsui M.T., Pan K. (2022). Effects and mechanisms of organic matter regulating the methylmercury dynamics in mangrove sediments. J. Hazard. Mater..

[32] Buchfink B., Xie C., Huson D.H. (2015). Fast and sensitive protein alignment using DIAMOND. Nat. Methods.

[33] Li L.G., Cai L., Zhang X.X., Zhang T. (2014). Potentially novel copper resistance genes in copper-enriched activated sludge revealed by metagenomic analysis. Appl. Microbiol. Biotechnol..

[34] Li L.G., Xia Y., Zhang T. (2017). Co-occurrence of antibiotic and metal resistance genes revealed in complete genome collection. ISME J..

[35] Cooper C.J., Zheng K., Rush K.W., Johs A., Sanders B.C., Pavlopoulos G.A., Kyrpides N.C., Podar M., Ovchinnikov S., Ragsdale S.W. (2020). Structure determination of the HgcAB complex using metagenome sequence data: Insights into microbial mercury methylation. Commun. Biol..

[36] Yin X., Jiang X.T., Chai B., Li L., Yang Y., Cole J.R., Tiedje J.M., Zhang T. (2018). ARGs-OAP v2.0 with an expanded SARG database and Hidden Markov Models for enhancement characterization and quantification of antibiotic resistance genes in environmental metagenomes. Bioinformatics.

[37] Alcock B.P., Raphenya A.R., Lau T.T.Y., Tsang K.K., Bouchard M., Edalatmand A., Huynh W., Nguyen A.V., Cheng A.A., Liu S. (2020). CARD 2020: Antibiotic resistome surveillance with the comprehensive antibiotic resistance database. Nucleic Acids Res..

[38] Arango-Argoty G.A., Guron G.K.P., Garner E., Riquelme M.V., Heath L.S., Pruden A., Vikesland P.J., Zhang L. (2020). ARGminer: A web platform for the crowdsourcing-based curation of antibiotic resistance genes. Bioinformatics.

[39] Feldgarden M., Brover V., Gonzalez-Escalona N., Frye J.G., Haendiges J., Haft D.H., Hoffmann M., Pettengill J.B., Prasad A.B., Tillman G.E. (2021). AMRFinderPlus and the Reference Gene Catalog facilitate examination of the genomic links among antimicrobial resistance, stress response, and virulence. Sci. Rep..

[40] Yang Y., Li B., Ju F., Zhang T. (2013). Exploring variation of antibiotic resistance genes in activated sludge over a four-year period through a metagenomic approach. Environ. Sci. Technol..

[41] Moore B. (1960). A new screen test and selective medium for the rapid detection of epidemic strains of Staphylococcus aureus. Lancet.

[42] Hintelmann H. (2010). Organomercurials. Their formation and pathways in the environment. Met. Ions Life Sci..

[43] Priyadarshanee M., Chatterjee S., Rath S., Dash H.R., Das S. (2022). Cellular and genetic mechanism of bacterial mercury resistance and their role in biogeochemistry and bioremediation. J. Hazard. Mater..

[44] Singh D.K., Lingaswamy B., Koduru T.N., Nagu P.P., Jogadhenu P.S.S. (2019). A putative merR family transcription factor Slr0701 regulates mercury inducible expression of MerA in the cyanobacterium Synechocystis sp. PCC6803. Microbiologyopen.

[45] Cardona G.I., Escobar M.C., Acosta-Gonzalez A., Marin P., Marques S. (2022). Highly mercury-resistant strains from different Colombian Amazon ecosystems affected by artisanal gold mining activities. Appl. Microbiol. Biotechnol..

[46] Tiwari A., Gomez-Alvarez V., Siponen S., Sarekoski A., Hokajarvi A.M., Kauppinen A., Torvinen E., Miettinen I.T., Pitkanen T. (2021). Bacterial Genes Encoding Resistance Against Antibiotics and Metals in Well-Maintained Drinking Water Distribution Systems in Finland. Front. Microbiol..

[47] Li X., Chen F., Chen Y. (2020). Gcluster: A simple-to-use tool for visualizing and comparing genome contexts for numerous genomes. Bioinformatics.

[48] Larsson D.G.J., Flach C.F. (2022). Antibiotic resistance in the environment. Nat. Rev. Microbiol..

[49] Rodriguez-Beltran J., DelaFuente J., Leon-Sampedro R., MacLean R.C., San Millan A. (2021). Beyond horizontal gene transfer: The role of plasmids in bacterial evolution. Nat. Rev. Microbiol..

[50] Mahbub K.R., King W.L., Siboni N., Nguyen V.K., Rahman M.M., Megharaj M., Seymour J.R., Franks A.E., Labbate M. (2020). Long-lasting effect of mercury contamination on the soil microbiota and its co-selection of antibiotic resistance. Environ. Pollut..

[51] Zhao Y., Hu H.W., Su J.Q., Hao X., Guo H., Liu Y.R., Zhu Y.G. (2021). Influence of Legacy Mercury on Antibiotic Resistomes: Evidence from Agricultural Soils with Different Cropping Systems. Environ. Sci. Technol..

[52] Pal C., Bengtsson-Palme J., Kristiansson E., Larsson D.G. (2015). Co-occurrence of resistance genes to antibiotics, biocides and metals reveals novel insights into their co-selection potential. BMC Genom..

